# A Cohort Perspective on the Demography of Grandparenthood: Past, Present, and Future Changes in Race and Sex Disparities in the United States

**DOI:** 10.1007/s13524-019-00795-1

**Published:** 2019-07-03

**Authors:** Rachel Margolis, Ashton M. Verdery

**Affiliations:** 1 Department of Sociology, University of Western Ontario, Social Science Centre 5326, London, Ontario N5A 5C2 Canada; 2 Department of Sociology and Criminology, Pennsylvania State University, 211 Oswald Tower, University Park, PA 16802 USA

**Keywords:** Grandparenthood, Historical demography, Population aging, Mortality, Fertility

## Abstract

How has the demography of grandparenthood changed over the last century? How have racial inequalities in grandparenthood changed, and how are they expected to change in the future? Massive improvements in mortality, increasing childlessness, and fertility postponement have profoundly altered the likelihood that people become grandparents as well as the timing and length of grandparenthood for those that do. The demography of grandparenthood is important to understand for those taking a multigenerational perspective of stratification and racial inequality because these processes define the onset and duration of intergenerational relationships in ways that constrain the forms and levels of intergenerational transfers that can occur within them. In this article, we discuss four measures of the demography of grandparenthood and use simulated data to estimate the broad contours of historical changes in the demography of grandparenthood in the United States for the 1880–1960 birth cohorts. Then we examine race and sex differences in grandparenthood in the past and present, which reveal declining inequality in the demography of grandparenthood and a projection of increasing group convergence in the coming decades.

**Electronic supplementary material:**

The online version of this article (10.1007/s13524-019-00795-1) contains supplementary material, which is available to authorized users.

## Motivation

The *demography of grandparenthood* refers to the prevalence, age patterns, and duration of grandparental roles as well as numbers of grandchildren among population members. It is an important demographic concept for understanding family dynamics and social inequality across generations because it determines the existence, timing, and extent of critical intergenerational relationships, which in turn condition many intergenerational transfers. Researchers have long recognized that being a grandparent offers benefits to older adults: grandchildren often provide important emotional meaning and support (Lye 1996; Silverstein and Long 1998). Likewise, grandparents can provide key inputs to grandchildren, aid adult children during periods when work and family demands are great, and can even decrease mortality of younger kin (Fuller-Thomson et al. 1997; Sear and Coall 2011; Yorgason et al. 2011). Understanding the demographic features of intergenerational relationships, how they have changed over time, and how they vary across groups is an integral part of contextualizing the social benefits associated with grandparenthood.

Grandparenthood, as a role and in its functions, tends to vary by sex and race. Women generally have children younger and live longer than men, making them more likely than men to experience grandparenthood for greater durations. Research on links between grandparental availability and child mortality has found sex differences in these associations, with the presence of grandmothers (especially maternal ones) being more strongly associated with positive grandchild outcomes than the presence of grandfathers (Sear et al. 2000; Sear et al. 2002). Likewise, researchers have long noted differences in the function and significance of grandparenthood between black and white families (Hunter and Taylor 1998), with qualitative work showing that grandparents provide exceptional levels of support in black families (Burton 1992,1996; Burton and DeVries 1992). Understanding how and why the demography of grandparenthood differs by sex and race will help to situate these findings within different demographic contexts.

Research about intergenerational relationships and transfers has often casually noted that their context depends on the demography of grandparenthood. However, most research on grandparenthood has relied on subpar, poorly defined measures that conflate age, period, and cohort, and it often lumps multiple cohorts or periods together. For example, researchers have used numerous measures of the timing of the transition to grandparenthood, including the average age of transition to grandparenthood among those with grandchildren (Szinovacz 1998), the age at which 50 % of all living older adults in a period are grandparents (Margolis 2016; Murphy 2011), and the average age at grandparenthood among a sample of those with a child at least 16 years old (Leopold and Skopek 2015a). Each of these measures suffers from several limitations. As period measures, they average over multiple cohorts, each has a different denominator, and each relies only on individuals who survive to the typical age at grandparenthood, which may obscure intercohort changes and the factors driving them. Even taken together, these measures offer an incomplete picture of the demography of grandparenthood. Moreover, the sheer variety of different measures impedes comparability across periods, populations, and studies.

Cohort measures of grandparenthood have important strengths compared with the more commonly estimated period measures. First, cohort measures are useful to researchers from multiple audiences who each can benefit from understanding different aspects of the demography of grandparenthood. These measures enable accounting for the different pathways to not experiencing grandparenthood, allowing researchers in different fields to study the factors most interesting to them. Gerontologists are likely interested in the proportion of those who reach adult ages that become grandparents, but perhaps not whether people do not become grandparents because they died early in life. Not experiencing grandparenthood because of early mortality, however, is a central insight for evolutionary demographers because it is a key mechanism by which an individual, or a set of individuals in a social group of interest, may not pass on his, her, or their genes. Family sociologists may be most interested in race and sex disparities in the timing and duration of grandparenthood rather than in the proportion who attain it. Cohort measures can provide detailed information about both central tendency and variation in these concepts. They can also highlight contemporaneous patterns in grandparenthood and the causes of race and sex differences. Last, cohort measures capture social changes in the meaning of grandparenthood and its timing. Peer groups are central to setting and regulating norms about family life (Ryder 1965), and cohort measures can capture changes in the norms of grandparenthood (e.g., when peers are transitioning into the role) that may in turn influence individual action (e.g., when older adults pressure their children to have children). However, to our knowledge, no research has elaborated a cohort perspective on grandparenthood or how it is changing.

In this study, we analyze simulated data to characterize broad types of historical changes in the demography of grandparenthood in the United States for the 1880–1960 birth cohorts. Taking a cohort perspective, we introduce the proximate determinants of grandparenthood and four measures of grandparenthood—the proportion becoming grandparents, age at transition to grandparenthood, length of grandparenthood, and number of grandchildren—that allow researchers from different fields to appreciate how demographic changes affect the demography of grandparenthood in ways most interesting to that field. We also investigate trends in sex and race differences in grandparenthood in the past, present, and near future, and the demographic processes shaping those inequalities. Our aim is not to provide accurate numbers by cohort, race, and sex, but rather to better understand the processes that shape changes over time and inequalities across groups in the demographic contours of grandparenthood. We conclude by reflecting on how a cohort perspective on the demography of grandparenthood compares with a period perspective and how our results might inform best practices for survey measurement of grandparenthood.

### The Proximate Determinants of Grandparenthood

A proximate determinants framework was first proposed to model the most important causes of fertility levels. As Bongaarts (1978:105) noted, “[s]ubstantial insight can be gained if, in addition to the socioeconomic factors influencing fertility, the specific mechanisms through which these factors operate are identified.” Similarly, the proximate determinants of grandparenthood offer a clear exposition of the determinants of the prevalence, timing, and duration of grandparenthood within a cohort or group by examining the important pathways through which people do not become grandparents. Like the proximate determinants of fertility, the proximate determinants of grandparenthood are predictable, and it is their predictability that makes them analytically useful. Future research can rely on this framework to make informed predictions about how grandparenthood will differ in different population subgroups, in different societies, and at different points in time.

Whether one becomes a grandparent^1^ and the timing of that transition depends on whether one has children, the timing of fertility, whether one’s children have children, their fertility timing, and mortality conditions. Taking a step back, grandparenthood depends on partnership and childbearing contexts, which are both indirectly related to marriage. These factors in turn affect the duration of grandparenthood. Grandparenthood has been called a “countertransition” (Hagestad 1988; Sprey and Matthews 1982) because it is determined by the actions of one’s children. To experience grandparenthood, one must have at least one child and live long enough to see that child have a child; from an evolutionary demography perspective, what matters is whether one’s child has a child, not necessarily whether one was alive when the event occurred. A cohort perspective allows us to contextualize both ways of seeing the phenomenon. We focus on the most important proximate determinants of these patterns, and we ignore factors that we expect play a much smaller role.^2^

The first pathway to not experiencing grandparenthood is one’s own childlessness.^3^ The cohort perspective on grandparenthood offers an important insight regarding this pathway by highlighting that within a birth cohort, some will die before reaching childbearing ages, whereas others will live through childbearing ages without bearing a child. Population-based surveys of adults will miss a substantial fraction of those who die before childbearing ages, affecting survey estimates of the size of this group, especially when child mortality is high. From an evolutionary demography perspective, cohort changes in child mortality are likely to be of greater interest than cohort changes in nonfertility as a mechanism affecting childlessness, given the magnitude of changes in these factors over time. From a family demography perspective, however, changes in nonfertility are likely to be the more interesting mechanism.

The second pathway to not experiencing grandparenthood is if all of one’s children remain childless for the duration of one’s life. As both family demographers and evolutionary demographers will recognize, the importance of this pathway will vary substantially with fertility levels: it is likely to affect few individuals when people have many children, and it will be more important when fertility is lower. As a simple example, a person with four children, each of whom has a 10 % probability of nonfertility, would have a 0.01 % expected probability of having no grandchildren, assuming independence, whereas a person with one child would have a 10 % probability. The cohort perspective would similarly note the role of premature mortality in differentiating this group into those whose children all die before childbearing ages, those whose children all survive childbearing ages without having borne a child, and those whose children all die during childbearing ages without having born a child. However, we do not make this distinction because the role of premature child death in affecting people’s chances of becoming a grandparent is likely to be miniscule given the fertility effects just discussed—that is, those with multiple children are exceedingly unlikely to see all of them die before childbearing ages—and because child mortality is generally low when fertility is low.

The third pathway to not experiencing grandparenthood is to die before one’s children have children. The likelihood of not experiencing grandparenthood because of this pathway is affected by the level of adult mortality in a society and the timing of grandparenthood’s onset, which is shaped by one’s own ages of childbearing as well as the ages of childbearing of one’s children. For evolutionary demographers and others concerned with genetic legacy, dying before one’s child has a child may be less relevant than it is for gerontologists and others who care about experiencing the meaning and opportunities associated with grandparental roles. No research has broken down the relative importance of these factors for explaining cohort experiences of grandparenthood, how they are changing over time, or how they vary across groups.

### Measures for the Demography of Grandparenthood

#### Measure 1: Proportion Who Have Grandchildren During Their Lifetime

The proportion of people in a birth cohort who have grandchildren during their lifetime represents how common it is to experience the role of grandparent and have the opportunity to experience a relationship with and make transfers to a grandchild. Demographers have long been interested in how demographic change affects what proportion of people overlap with different types of kin (Watkins et al. 1987; White and Preston 1996). The proportion of people who experience grandparenthood during their lifetime is by definition a cohort measure that requires data over the entire period when members of the cohort are alive. Because these data are rarely available, very few studies estimate this measure.

Some research has used demographic microsimulation or models from mathematical demography that allow for cohort interpretations of grandparenthood, but we have been able to locate only one working paper that presents cohort estimates of the lifetime prevalence of experiencing this role (Chung 2017). Instead, studies using microsimulation or mathematical demography typically focus on mean counts of grandchildren (see the upcoming discussion of “grandparity”). Other work in these computational and mathematical traditions has not looked at grandparenthood (i.e., individuals who have grandchildren), instead focusing on whether people have living siblings, parents, children, cousins, or grandparents (e.g., Le Bras 1973; Uhlenberg 1996; Verdery 2015; Watkins et al. 1987).

Other research has used survey data to estimate the proportion of adults of a certain age in a certain year who are grandparents (e.g., Dykstra et al. 2006; Kemp 2003; Puur et al. 2011; Skopek and Leopold 2017; Szinovacz 1998). These studies have often estimated the proportion of grandparents among people reaching an age that is thought to be the typical age of grandparenthood, a measure of interest to many family sociologists and gerontologists. However, these studies presented a variety of estimates that are difficult to compare. An example is Szinovacz (1998), who used data from the 1992–1994 National Survey of Families and Households (NSFH) and noted that most individuals will experience grandparenthood. Szinovacz reported that 20 % to 33 % of adults in the survey have grandchildren, 66 % of adults with children older than age 15 have grandchildren, and 95 % with children above age 40 are grandparents. The choice of denominator matters greatly for estimates of the prevalence of grandparenthood.

Although survey estimates can be informative, they are not cohort measures and should not yield cohort interpretations. For one, they do not account for mortality before respondents reach the ages of survey eligibility, missing an important evolutionary mechanism. Second, such measures average across multiple birth cohorts to get a period prevalence for a large age group because sample sizes in many surveys are limited. Averaging across birth cohorts in this fashion precludes studying changes in grandparenthood over time, which might mask important trends in inequality of interest to family sociologists. Third, the data are censored, meaning that some people are without grandchildren at the time of the survey but will become grandparents later in life. Such data censoring can bias understandings of reproductive success, especially because period shifts in fertility timing (tempo) can distort synthetic cohort interpretations of the level of fertility (quantum) (Bongaarts and Feeney 1998). The final limitation of period measures of grandparenthood is that they preclude studying its proximate determinants and the factors behind subgroup differences in grandparenthood.

#### Measure 2: “Grandparity,” or Number of Grandchildren

Estimates of the number of grandchildren have been extensively studied (e.g., Goodman et al. 1974; Murphy 2011; Puur et al. 2011; Szinovacz 1998). We refer to the number of grandchildren people have during their lifetime as “grandparity.” This measure is useful because it represents the number of grandparent/grandchild relationships that one can have. However, researchers from different disciplines conceptualize and measure grandparity differently, depending on their motivation. Biologists and evolutionary demographers are interested in the number of grandchildren ever born to the individual as well as whether grandchildren are born while the person is alive because this number is a measure of reproductive success (Bolund and Lummaa 2017; Goodman and Koupil 2009; Goodman et al. 2012). Those examining lineages use similar measures of number of descendants (Kolk and Hällsten 2017) or patrilineal descendants (Song et al. 2015). These studies relied on historical genealogical data, register data, or historical church records (Chapman et al. 2017; Song and Campbell 2017). In general, few data sets from the United States are up to the task of analyzing these issues, and those that are available either are limited to highly unique populations (e.g., Utahns) or cover only relatively recent birth cohorts (e.g., those in the Panel Study of Income Dynamics, Wisconsin Longitudinal Study, or National Longitudinal Study of Youth; see Song and Campbell 2017).

Other work used demographic microsimulation or mathematical modeling to estimate the population average numbers of living grandchildren for adults of different ages. This measure gives a sense of how average numbers of living grandchildren vary across ages, especially for ages at which grandparenthood is common. Unlike total ever born, the number of grandchildren alive at different ages is particularly interesting to family demographers and gerontologists. Some work in this tradition includes the results presented by Murphy (2011), who reported the average number of living grandchildren around age 75 for different birth cohorts in Britain, and mathematical demographic analyses by Goodman et al. (1974), who reported mean numbers of granddaughters ever born and still living for women at ages 30 to 85 according to stable population models of 1960s vital rates.

Empirical work based on survey data offers a more confusing picture. The most common measure examined in such studies is the mean number of grandchildren among those with any grandchildren. However, as with measures of the prevalence of grandparenthood, it is often challenging to tell who is included in the denominator. For example, Szinovacz (1998) noted that the mean number of grandchildren for all adults in the 1992–1994 NSFH was 5.5 when self-defined grandchildren were included and a little lower, 5.2, when only children’s children were included. However, in this study, it is not clear whether this mean is estimated only among those with any grandchildren, or whether the mean includes those with none. Other studies are clearer and reported the distribution of numbers of grandchildren in addition to the mean numbers (Daw et al. 2016; Puur et al. 2011). However, even the clearest estimates from survey data are limited by left- and right-censoring.

#### Measure 3: The Timing of the Transition to Grandparenthood

The transition to grandparenthood is marked by the age at which people have their first grandchild. Most research based on microsimulation or mathematical demography has not examined this (except the working paper by Chung 2017), but it is a commonly noted measure in research using survey data. However, there is no clear agreement about how it should be estimated. For example, if a survey includes information on the timing of grandparenthood among grandparents, then the average age of transition to grandparenthood can be reported, averaged over cohorts (Szinovacz 1998). Another measure is the age at which one-half of older adults in a period are grandparents, which averages over cohorts but includes everyone in the denominator who survives until those years (Margolis 2016). Other measures of the transition to grandparenthood include the age at which 50 % of those who had children at the time of the survey became grandparents (Leopold and Skopek 2015a) or the average age at grandparenthood among a sample of those with a child at least 16 years old (Leopold and Skopek 2015b). Nonetheless, each measure suffers the limitations of period measures described earlier: they do not account for left-censoring, and they preclude decomposition into the proximate determinants of grandparenthood. Moreover, the sheer variety of different measures simultaneously fails to offer a complete picture of this phenomenon and also impedes comparability across periods, populations, and studies. How should we measure the transition to grandparenthood? Ideally, we would measure both central tendency (e.g., medians or means) and dispersion (e.g., interquartile ranges or standard deviations) of the distribution in the timing of grandparenthood transitions among those who ever become grandparents—a series of cohort measures.

#### Measure 4: The Duration of Grandparenthood

How long are people grandparents? This aspect of the demography of grandparenthood has received the least scholarly attention, but it may be the most consequential when considering multigenerational effects and how the extent of intergenerational relationships might condition intergenerational transfers. Cohort measures of the duration of grandparenthood are desirable for understanding family relationships. Of interest is the duration of grandparenthood among those who experience the role, which necessitates knowing when people first become grandparents and when they die. However, such measures are rarely available because few surveys follow respondents until death, and fewer still ask about the age at transition to grandparenthood, both of which must be known to estimate the duration of grandparenthood among those who experience this role. Only two studies reported true cohort measures for the duration of grandparenthood: a recent one using unique church record data from Finland (Chapman et al. 2017) and a microsimulation (Chung 2017).

In the absence of cohort measures, recent studies have begun to examine period approximations of the duration of grandparenthood. In a first attempt at examining how the duration of grandparenthood varies across contexts, Leopold and Skopek (2015b) subtracted the median age of the transition to grandparenthood from period life expectancy across OECD countries to operationalize the concept. This procedure yields plausible estimates across countries, but it is unclear how to interpret them. Other work estimated expected years of grandparenthood remaining by drawing on cross-sectional data and health expectancy methods (Margolis 2016; Margolis and Wright 2017; Yahirun et al. 2018). These measures estimate the mean duration of grandparenthood for a hypothetical cohort that experiences the period grandparenthood prevalence, health, and mortality conditions. Period estimates of the duration of grandparenthood, like period estimates of life expectancy, give an average length of grandparenthood for a synthetic cohort of population members. They have a direct demographic interpretation and can contextualize macro-level changes in the length of grandparenthood over time or across groups. However, they also suffer limitations. One key limitation is that these measures provide the average expected duration of grandparenthood for the whole population, including those who never become grandparents. These period estimates reveal nothing about the length of grandparenthood among those who become grandparents, which is needed for understanding the period of overlap for family relationships, intergenerational transfers, and multigenerational effects. They also focus on averages and ignore dispersion, which may exhibit interesting variability that maps onto sociodemographic groups of interest.

### Sex and Racial Inequality in Grandparenthood

Many studies have documented differences between men and women and between blacks and whites in the United States in different aspects of the demography of grandparenthood. Because of race and sex differences in the timing of fertility, blacks and women have been shown to transition to grandparenthood earlier than whites and men in the United States (Daw et al. 2016; Cherlin and Furstenberg 1986; Chung 2017; Szinovacz 1998). Black Americans also seem to have slightly more grandchildren than white Americans (Daw et al. 2016; Szinovacz 1998). As for the duration of grandparenthood, period estimates from Margolis and Wright (2017) found that women spend more years as grandparents than men, and whites spend more years as grandparents than blacks; microsimulation work containing cohort estimates yields the same interpretation (Chung 2017).

How and why are racial inequalities likely to change in the future? No published work has addressed this. Chung’s (2017) working paper showed that differences in the timing of grandparenthood are likely to grow but that the duration of grandparenthood may become more equal in the future. Projections by the U.S. Census Bureau postulate trends toward convergence in fertility and mortality rates for black and white Americans (Colby and Ortman 2014; U.S. Census Bureau 2012), although they suggest persistent disparities between groups in terms of these rates even as late as 2060. When fertility and mortality projections are examined in isolation, it is unclear whether race disparities in the demography of grandparenthood will shrink or persist in the coming decades even if the projections are correct.

## Data and Method

Microsimulation has a long history in demography and is generally considered the best approach for analyzing the relationship between changes over time in demographic rates and the availability of family and kin (Murphy 2011; Wachter, Blackwell, & Hammel 1997). Other approaches to studying the relationship between demographic rates and family availability are limited by the requirements of stable population models (e.g., Goodman et al. 1974), such as the assumptions of unchanging vital rates or single-sex models. More complex mathematical demographic methods are available that account for changing vital rates (Keyfitz and Caswell 2005), but they do not typically incorporate the complexity needed to model the multifaceted nature of demographic change; for instance, there are no readily available methods for incorporating the parity structure of fertility into mathematical models of kinship. As Keyfitz and Caswell noted (2005:388), such parameters can be added to mathematical demographic models, but the resultant calculations are “awkward enough that no one is likely to do it.” Parity structure can be incorporated into life tables or multistate cohort component demographic models (Keyfitz and Caswell 2005:573), but such models are inadequate for understanding kinship because they are not analytically tractable tools for modeling what happens to both members of a kin pair. For questions such as the changing demography of grandparenthood in the United States, mathematical approaches are much more limited than microsimulation.

Demographic microsimulation simulates the behaviors and circumstances of synthetic individual agents. Each agent experiences different demographic events over time—childbearing, marriage, divorce, remarriage, and death—with the likelihood of each event’s occurrence defined probabilistically by period according to static and dynamic agent attributes. As agents age, their probabilities of bearing a child, marrying, and dying change according to age-specific patterns. Such patterns differ by sex and other ascribed attributes—in our case, race. They also differ by achieved and changing characteristics, including number of prior children (parity) and marital status (e.g., we model nonmarital fertility). These probabilities vary across periods. We use “closed” microsimulation models, wherein marriages take place between two simulated agents, rather than “open” models, wherein each marriage coincides with the creation of a new agent (Van Imhoff and Post 1998). These procedures enable us to trace the hypothetical population history as well as the evolving kinship networks of simulated individuals. After the simulation, the resultant data are like a census with perfect information on each agent’s time of birth, death, childbirth, marriage, divorce, remarriage, and other demographic characteristics. It is also like a saturated or “sociocentric” network sample of the kinship network (Perkins et al. 2015; Verdery et al. 2012).

We use data from a previously reported and validated demographic microsimulation that examined the historical, contemporary, and projected prevalence of older adults without living family members in the United States from 1880–2060 (Verdery and Margolis 2017).^4^ In the online appendix, we present additional details about this microsimulation, including an overview of data inputs and comparisons of the simulated results to empirical estimates from surveys and other sources. Our analyses in this article focus on the demography of grandparenthood, which has not been examined in prior research using these data. This microsimulation used the Berkeley Socsim model, which is freely available to researchers (Hammel 1976; Hammel et al. 1990; Socsim 2016).

Our results reflect population and kinship dynamics for single-race, native-born, non-Hispanic black and white individuals. These are the largest race groups in the United States, especially when considering older adults, accounting for 84 % of the population ages 50 and older in 2014 and projected to account for 63.3 % of those ages 50 and older in 2060 (U.S. Census Bureau 2014). Separate models are estimated for black and white individuals to more faithfully model within-race similarities in demographic behaviors, which if ignored, can bias estimates of the distribution of kin (Ruggles 1993). Although racial intermarriage rates are increasing among younger generations, our focus on grandparents—unlikely to identify with two or more races—guides our choice to not model intermarriage (U.S. Census Bureau 2014). Likewise, we do not model immigration or emigration because microsimulation is not well suited to modeling immigration dynamics or migrant groups. Socsim does not allow people to age beyond 100 years, which may affect our estimates of the grandparenthood’s duration but only slightly: the updated Coale-Demeny Model West life tables suggest that only 0.07 % of women will survive to age 100 when life expectancy at birth is 60 and that those who do would have an expected 1.8 years remaining; when life expectancy at birth is 80, these numbers are 1.46 % and 2.2 years remaining, respectively. Although these limitations constrain the generality of our findings, we are constrained by the limits of microsimulation approaches.

Microsimulations are approximations. They ignore many relevant population features (Ruggles 1993), which can bias the dispersion of estimates (e.g., how many people have few children compared with many) but does not typically affect mean estimates. Modeling parity-specific fertility transitions, including childlessness, as we do, helps overcome some of these limitations. Ultimately, though, any microsimulation is more useful for understanding broad trends than making specific estimates. Recognizing these issues, we restrict our interpretations to general statements presented graphically and note that the results presented are simulation estimates, not based on empirical data.

## Results

**Fig. 1:**
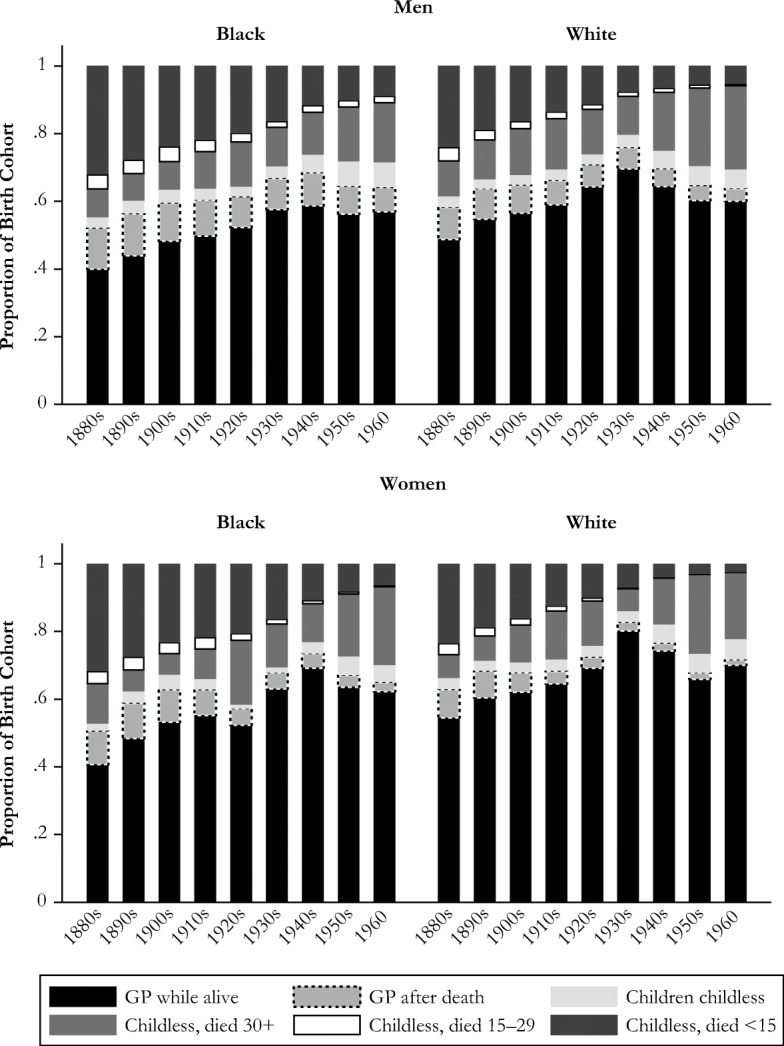
Experience of grandparenthood by race and sex: Men (panel a) and women (panel b) born in 1880–1960.

First, we examine the proportion of people across birth cohorts who become a grandparent during their lives and, if not, why (Fig. 1). The black bars at the bottom show that the percentage of adults who have grandchildren during their lives has increased from 40 % to 54 % for those born in the 1880s to highs of about 60 % of black men, about 70 % of white men and black women, and about 80 % of white women for the cohorts born in the 1930s and 1940s.

Figure 1 further breaks down the reasons that people do not have grandchildren while they are alive. The gray bars (just above the black ones) represent the proportion of birth cohorts whose children have children after they die. This represents a group that evolutionary demographers should care about because this group of deceased individuals pass along their genes even if they do not get to experience the grandparenthood role. As adult mortality has declined, this has become a less important reason for not experiencing grandparenthood over the period of analysis, decreasing by approximately one-half (from about 1 in 10 people to less than 1 in 20) for all race and sex groups except black men, for whom it is a little higher. The light gray bars represent people who do not become grandparents because all their children remain childless, and this factor has approximately doubled (albeit from low levels of about 3 %) over time because more recent birth cohorts have fewer children, making the likelihood that all of one’s children are childless at the time of one’s death higher today than it was in the past. The predominant reason across all birth cohorts for not becoming a grandparent is one’s own childlessness (shown in the three stacked portions of the bars at the top of the figure’s range). We distinguish between those who do not become a grandparent because of mortality at young ages (ages <15), those who reached childbearing ages but not the age of grandparenthood (ages 15–29), and those who survived to the age when they could become a grandparent but never did (died at age 30 or above). Figure 1 highlights the massive decline in young adult mortality (ages <15 and 15–29) and large increase in one’s own childlessness upon reaching age 30.

Among both blacks and whites, the levels of eventual grandparenthood are higher for women than for men. For example, for whites born between the 1880s and 1910s, about 5 % more white women became grandparents than white men. Sex differences in eventual grandparenthood for whites peaked for the 1930s and 1940s cohorts and then declined. Among blacks, women and men born in the 1880s and 1890s were similarly likely to become grandparents; sex differences increased, reaching 10 % for the 1940s birth cohorts, and then declined.

**Fig. 2:**
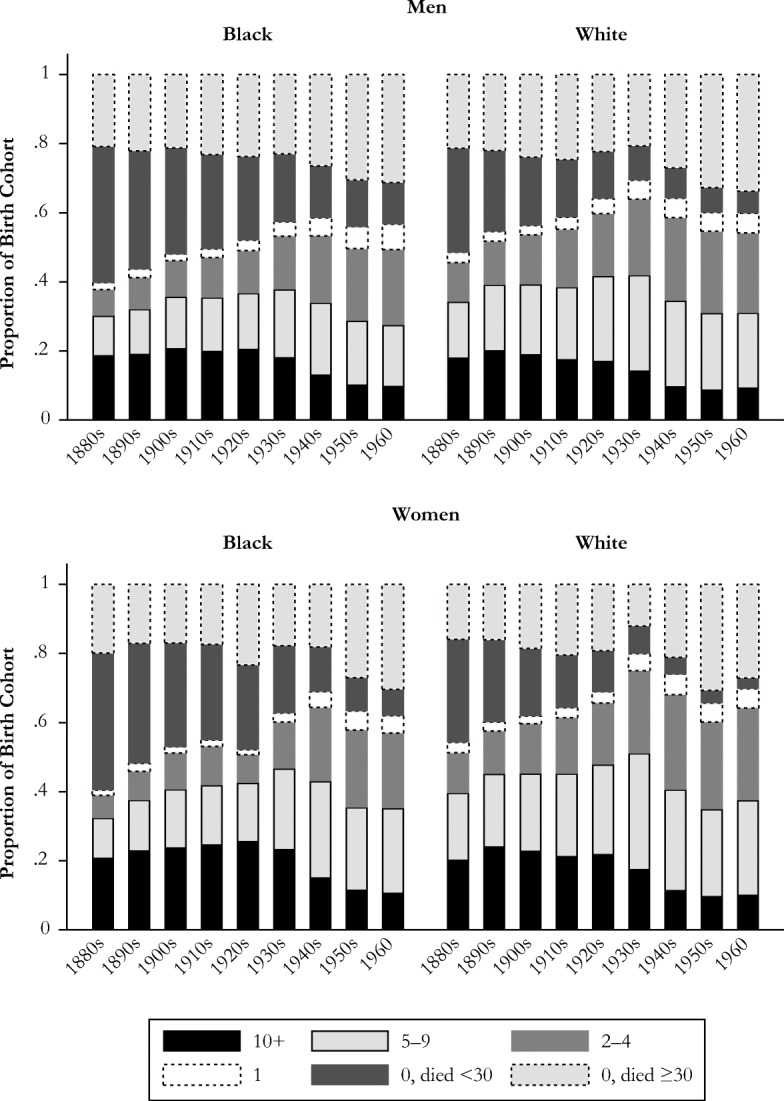
Number of grandchildren born while alive, by race and sex: Men (panel a) and women (panel b) born in 1880–1960.

Figure 2 charts the number of grandchildren born during one’s lifetime across birth cohorts by sex and race. The black bars at the bottom show that a decreasing proportion of people will have 10 or more grandchildren. The three sets of bars above that show that it is becoming much more common for people to have 5–9 grandchildren, 2–4 grandchildren, or only 1 grandchild. The top two bars show the proportion who do not have grandchildren while they are alive, breaking up the group who reach the age at which they could become a grandparent and those who die before age 30. We also see from Fig. 2 that women tend to have more grandchildren than men, mostly due to higher rates of ever becoming a parent and, hence, a grandparent. Still, the changing distribution of number of grandchildren is similar across race and sex groups.

**Fig. 3:**
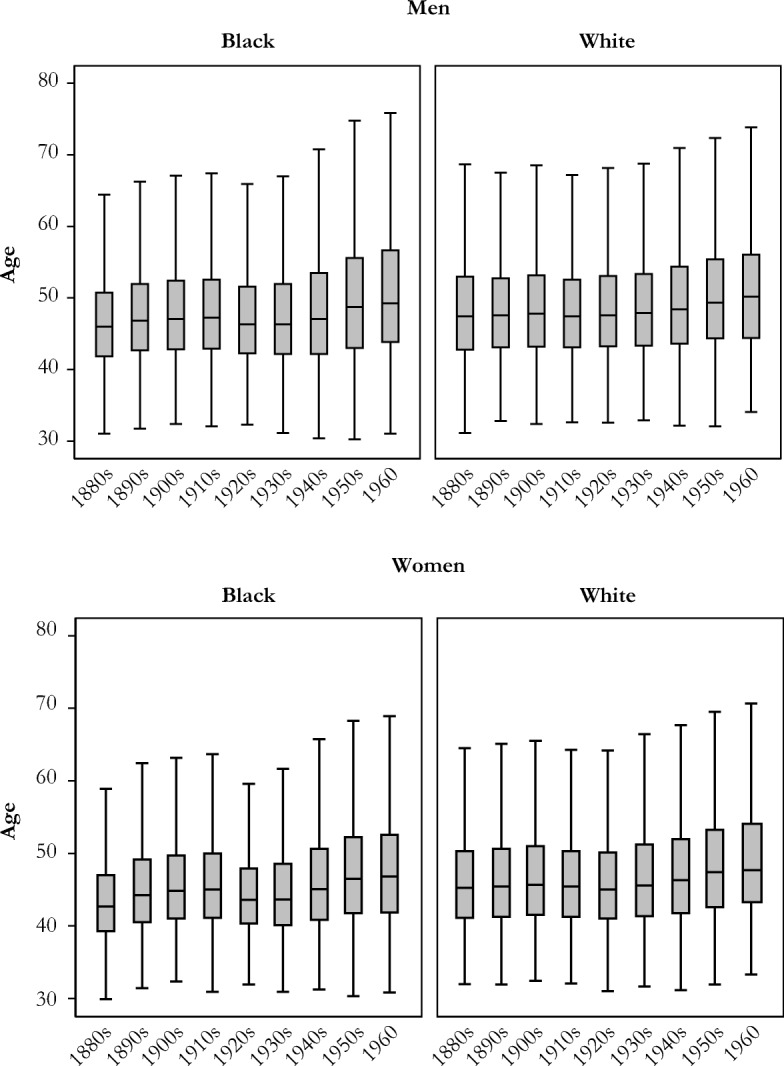
Age at first grandchild, by race and sex: Men (panel a) and women (panel b) born in 1880–1960.

Next, we consider the age at the transition to grandparenthood among those who experience this role (Fig. 3) using box and whisker plots that capture important features of the distribution of these transition timings.^5^ Among those who become grandparents during their lifetime, the median age at becoming a grandparent has increased for both women and men over time. For white women born in the 1880s, the median age was in the mid-40s. It was a few years (2.5) younger for black women. It increased by a few years (2.5) for white women and by more years (4) for black women over the period of our analysis. Among white and black men, the median age at grandparenthood was in the upper 40s for the 1880s cohorts. The median age increased by a few years (3) for both groups over the period of our analysis. A focus on the central tendencies, however, obscures an important point: variation in the age at grandparenthood has also increased dramatically over time, with larger increases for blacks than for whites.

**Fig. 4:**
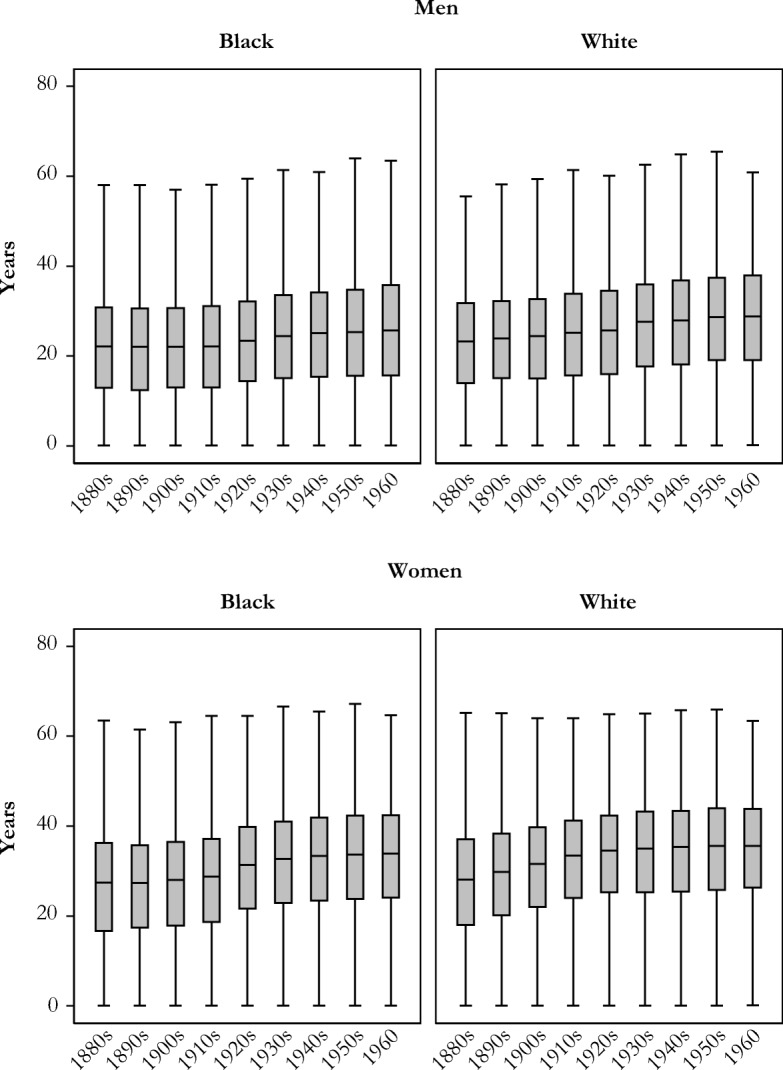
The duration of grandparenthood among those who become grandparents during their lifetime, by race and sex: Men (panel a) and women (panel b) born in 1880–1960.

The duration of grandparenthood has also increased over time, despite a later transition to grandparenthood. Figure 4 plots variation in the duration of grandparenthood among those who become grandparents during their lifetimes. Despite delayed grandparenthood, declines in older adult mortality have increased the median length of grandparenthood for white and black men and women over time. These changes, however, are not uniform: women saw larger increases than men, and whites saw larger increases than blacks. Our simulation estimates indicate that the median length of grandparenthood increased from 22 to 26 years for black men (four years), 27 to 34 years for black women (seven years), 23 to 29 years for white men (six years), and 28 to 36 years for white women (seven years). In addition to longer periods of grandparenthood for all race and sex groups, there are also increases in the variation of this measure for each group. This is an important finding because it implies that there is increasing inequality in multigenerational overlap within as well as between sex and race groups.

To understand the demographic forces shaping the duration of grandparenthood, we turn to Table 1. The timing of grandparenthood is determined by one’s age at first birth and the first birth of any child’s child. We present the means of these ages in the simulated data within different birth cohorts in the first two rows. Over the period of our simulation, the mean age at first birth for grandparents increased, as did the mean age of one’s child at the birth of the first grandchild, leading to an increase in the age at the transition to grandparenthood. We also show trends in increasing mean age at death for select cohorts, showing evidence of mortality decline. The mean length of grandparenthood can be shown as the difference between the mean age at grandparenthood and mean age at death.

**Table 1:** Demographic factors affecting the duration of grandparenthood, by race, sex, and select birth cohorts, estimated from simulated data

	Birth Cohort				
	1880s	1900s	1920s	1940s	1960
White Men					
Mean age at first birth	25.0	25.4	25.3	25.2	26.0
Mean age of child at birth of first grandchild	23.3	23.2	23.3	24.4	24.8
Mean age at grandparenthood	48.3	48.6	48.6	49.5	50.8
Mean age at death	71.5	72.7	73.9	77.1	79.1
Mean length of grandparenthood	23.1	24.1	25.3	27.6	28.3
Black Men					
Mean age at first birth	24.3	25.1	24.8	25.0	26.0
Mean age of child at birth of first grandchild	22.5	23.1	22.8	23.6	24.7
Mean age at grandparenthood	46.8	48.2	47.6	48.5	50.6
Mean age at death	68.8	70.4	71.1	73.4	76.5
Mean length of grandparenthood	22.1	22.1	23.5	24.9	25.8
White Women					
Mean age at first birth	22.9	23.2	22.8	23.0	23.9
Mean age of child at birth of first grandchild	23.5	23.5	23.5	24.4	25.0
Mean age at grandparenthood	46.3	46.7	46.3	47.4	49.0
Mean age at death	73.7	77.2	79.5	81.5	83.4
Mean length of grandparenthood	27.4	30.5	33.3	34.0	34.5
Black Women					
Mean age at first birth	21.2	22.8	21.8	23.1	23.6
Mean age of child at birth of first grandchild	22.6	23.3	22.9	23.3	24.3
Mean age at grandparenthood	43.8	46.1	44.8	46.4	47.8
Mean age at death	70.3	73.1	75.2	78.7	80.8
Mean length of grandparenthood	26.5	27.0	30.4	32.3	32.9

*Note:* Summary measures in this table include only people who became grandparents during their lives.

**Fig. 5:**
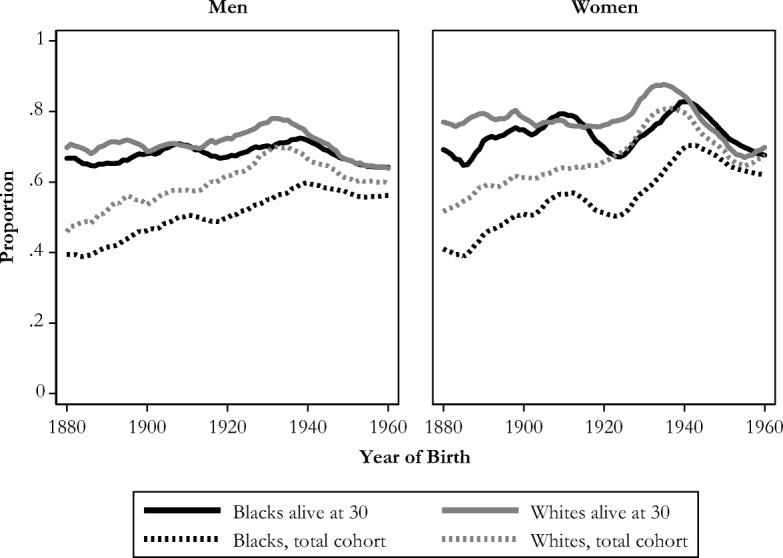
Proportion becoming a grandparent while alive, by race and sex. Shown with five-year moving averages.

Next, we summarize trends in racial inequality in grandparenthood by plotting race differences by sex side by side.^6^ First, Fig. 5 highlights that historically, for the 1880s–1940s cohorts, whites were much more likely to become grandparents than blacks, but racial differences are negligible for recent cohorts. However, when we plot the proportion becoming grandparents only among those who survive to age 30, we see that the long-standing race differences in eventual grandparenthood are negligible, highlighting that blacks’ higher rates of mortality at young ages accounts for the white advantage in becoming a grandparent across most cohorts. This figure highlights surprising continuity in grandparenthood over time, conditional on survival to age 30. Despite massive shifts in life expectancy and fertility, fluctuations in grandparenthood among adults has been remarkably stable.

**Fig. 6:**
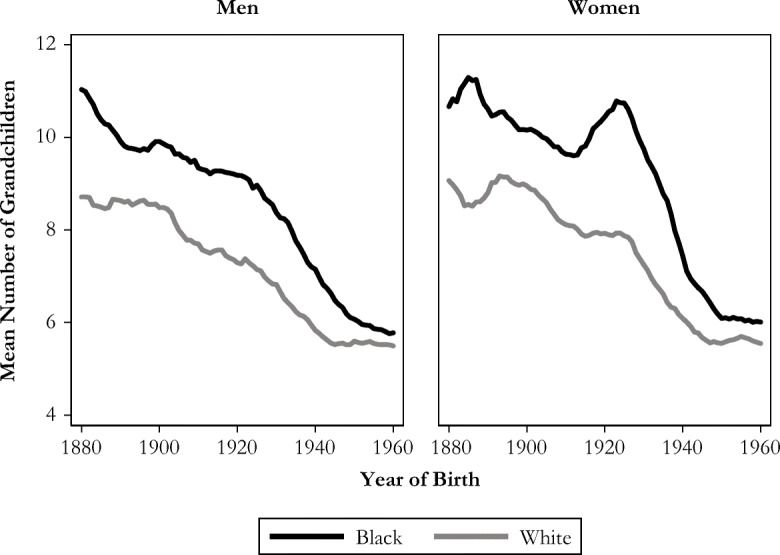
Mean number of grandchildren while alive among those who become grandparents, by race and sex. Shown with five-year moving averages.

Second, Fig. 6 shows that historically, blacks had about two more grandchildren than whites. Racial differences are largest among those born in the 1920s but have all but disappeared for recent cohorts.

**Fig. 7:**
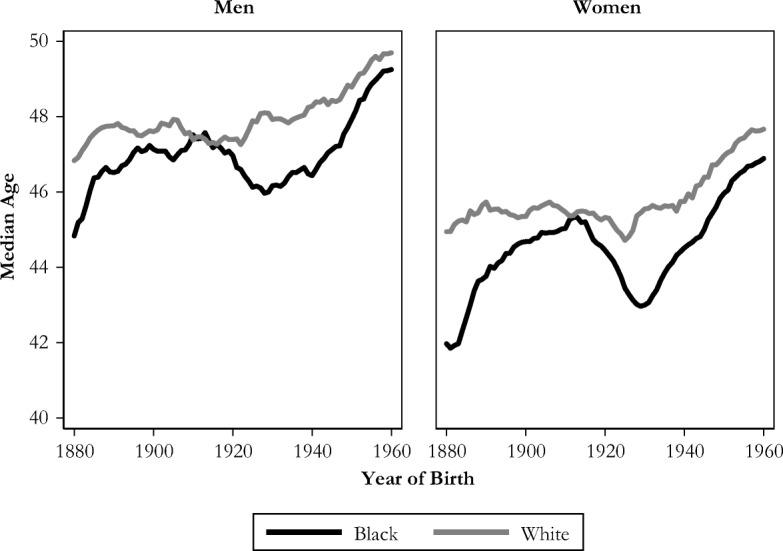
Median age at first grandchild among those who become grandparents, by race and sex. Shown with five-year moving averages.

Third, racial differences in the timing of grandparenthood have also decreased over time, from about a five-year gap in the median age at the transition to grandparenthood for those born in the 1880s (blacks earlier than whites) to less than one year for recent cohorts (Fig. 7).

**Fig. 8:**
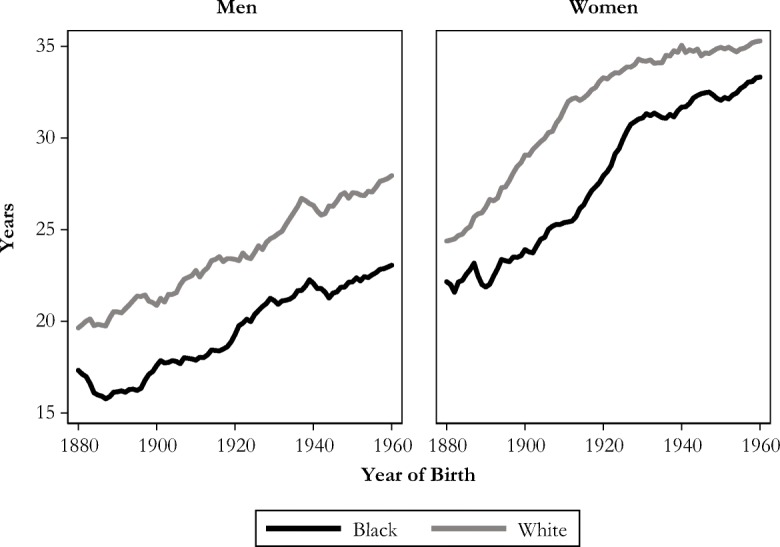
Median length of grandparenthood among those who become grandparents, by race and sex. Shown with five-year moving averages.

Last, the one measure for which we do not see convergent trends is the length of grandparenthood, especially among men: white men continue to have a few more years of grandparenthood than black men (Fig. 8). In summary, although historical cohorts had large racial inequality in the demographic experience of grandparenthood, racial gaps in three of our four measures have decreased substantially and are small for recent cohorts.

We also examine whether grandparents had grandchildren through their sons or daughters. Research has noted large differences between maternal and paternal grandparents for the strength and type of the grandparent-grandchild relationship (Mueller and Elder 2003; Uhlenberg and Hammill 1998), coresidence (Pilkauskas and Martinson 2014), and effects on grandchild survival (Jamison et al. 2002; Sear and Mace 2008). Although previous research has often noted earlier transitions to grandparenthood among grandmothers (Hagestad 1986; Sprey and Matthews 1982), we capture for the first time the duration of grandparenthood from sons compared with daughters. We find that the length of overlap with grandchildren from daughters is generally a few years longer than with grandchildren from sons. Thinking in terms of proximate determinants, this result owes to the younger average and lower variance in ages of first childbearing for women than men, a nearly universal pattern. The size of this gap is stable across birth cohorts, sex, and race. We posit that longer durations of maternal grandparenthood may play a role in explaining the more robust associations between presence of maternal grandparents—especially maternal grandmothers—and grandchild outcomes, either directly through increased exposure or indirectly through greater planning and investment in such a role.

## Discussion

Our analysis of the demography of grandparenthood takes a cohort perspective to outline the proximate determinants of grandparenthood and examines how the demographic contours of grandparenthood in the United States have shifted over a period of massive improvements in survival, fertility decline, and fertility postponement. Our findings demonstrate the factors behind later and longer grandparenthood and highlight a potential racial convergence in most features of its demography.

The proximate determinants of grandparenthood are a way to examine the pathways through which people do not become grandparents and the proportion of individuals in a cohort who do become grandparents. Our results highlight large changes in the reasons why people do not become grandparents during their lifetime and provide insight grandparenthood might continue to change in the future. There have been large declines in the proportion who do not become grandparents because of early mortality. For the 1960 birth cohort, mortality before age 30 accounts for fewer than 1 in 20 of those who do not become a grandparent for black women and white men and women, showing that little additional declines are expected for these groups. However, for black men, 1 in 10 does not become a grandparent because of mortality before age 30, highlighting the potential increase in grandparenthood with mortality declines at younger ages. We have seen large increases in the proportion who do not become a grandparent because of nonfertility: they have reached the age when it is possible to become a grandparent but die childless, or all their children remain childless. These groups together now make up 25 % to 30 % of the 1960 birth cohort, and this group is likely to continue to increase as nonfertility increases.

Our cohort estimates of the proportion becoming grandparents can put published period estimates from survey data into context. For example, examining long-run historical change, we can see that it is only a relatively recent phenomenon that “most individuals will experience grandparenthood” (Szinovacz 1998:40). When interpreting the prevalence of grandparenthood from recent population surveys, we must remember that the period prevalence is subject to both left- and right-censoring. Later and more variable transitions to grandparenthood mean that right-censoring of grandparenthood in population surveys will become more common as time goes on. We hope that future research can use the proximate determinants of grandparenthood to predict how grandparenthood will differ in different population subgroups, societies, and periods.

Race and sex differences in kinship structures, functions, and meaning are key topics for family sociologists (e.g., Burton 1992, 1996; Cherlin and Furstenberg 1986; Daw et al. 2016; Stack 1974), and the changing demography of grandparenthood can have implications for this literature. For example, estimates of longer durations of grandparenthood for women than men, and for whites than blacks, showcase changes in the period when multigenerational transfers can be made and relationships are developed. These estimates offer a demographic hypothesis for why grandmothers are regularly found to have greater effects on grandchild survival and well-being than grandfathers, and for why maternal grandmothers feature so prominently in intergenerational research. Using our simulated data, we can examine projected changes in inequality in grandparenthood through the 1960 birth cohort, adults who are now approaching retirement ages. Our results show projected convergence in three main aspects of the demography of grandparenthood for blacks and whites: (1) the proportion becoming a grandparent while alive, (2) the mean number of grandchildren among those who become grandparents, and (3) the median age at first grandchild. The one aspect where we see no sign of racial convergence is the length of grandparenthood. Even with projected trends toward racial convergence in mortality rates (Colby and Ortman 2014), our results point to persistent inequality in overlap between grandparents and grandchildren by race, which affects transfers and time for these relationships.

Given that most family demography relies on population surveys, we offer some ideas for how researchers can try to reconcile survey measures with cohort understandings, or at least examine the strengths and weaknesses of what can be measured. First, with survey data, one can measure the proportion of an age group, or the surviving members of a cohort, who are grandparents and, if they are not grandparents, whether it is due to their own childlessness or their children’s childlessness. Researchers can be clear about who is in the denominator: all older adults (ideal), just parents, or parents with children of a certain age. Researchers should also acknowledge that data are often right- and left-censored. A second measure is the number of grandchildren. Researchers should note whether the mean or median is estimated and whether the figures include people with no grandchildren or only those with at least one grandchild. Ideally, researchers should focus on the number alive for adults in relatively narrow age ranges, such as ages 70–74, and should also note limitations by left- and right-censoring. To capture the age at the transition to grandparenthood, few surveys collect retrospective data about the start of grandparenthood. And even with such data, we still have right- and left-censoring. With period data, the best measure would capture the age at which 50 % of individuals in a cohort have grandchildren, which can be compared with other data sources. Last, the length of grandparenthood can be best captured with cohort measures (as we have done) but can also be compared with period expectancies. One main difference between these measures is that with cohort data, we estimate the duration of grandparenthood among those who experience it, whereas period expectancies average over the whole population, including those who do not become grandparents. This explains why the estimates for period expectation of grandparenthood conducted by Margolis and Wright (2017) (21.5 years for men and 25.5 years for women in 2010) are lower than those presented here.

This analysis also has limitations. First, our focus on the demography of grandparenthood excludes grandparent-grandchild relationships, degree of contact, geographic proximity, and emotional closeness. Second, we do not consider step-grandparenthood here, but other work has estimated this from a period perspective (Yahirun et al. 2018). It is important to keep in mind that our results reflect single-race, native-born, non-Hispanic black and white individuals, ignoring migration and mixed-race unions. Thus, simulated cohort data and the contemporary U.S. population will always differ. Our simulation data fit population-level indicators of fertility, mortality, and population size, but there are nuanced components of demographic history that we did not examine. For instance, we suspect that our estimates of grandparity, especially at higher levels, may be off slightly because the simulation data we use came from models that did not distinguish rates of third births from rates of higher-order births; relatedly, we did not model intergenerational correlations in fertility (Ruggles 1993). However, even if the precise grandchild counts, for instance, are not quite right, the trends that we study are likely to be valid approximations. In addition, because our simulated population cannot age past 100 years, our estimates for the duration of grandparenthood for later cohorts may be slightly underestimated. Simulation analysis is not well suited to examine differences in grandparenthood by socioeconomic status—for instance, because the data are often unavailable and it is challenging to model transitions between groups—but research using other methods could examine this. Last, future research could consider these processes in longer historical perspective or in other countries. It may be particularly interesting to look at grandchildlessness in lowest-low fertility contexts.

Grandparents are thought to play an important role in the transmission of advantage in families and in social mobility and stratification (Mare 2011,2014; Song 2016; Zeng and Xie 2014). Much research has documented grandparental effects on grandchild well-being, cognitive achievement, and personal development (Chan and Boliver 2013; Silverstein and Ruiz 2006). The demography of grandparenthood can help to contextualize this research, offering a simple analytical framework for understanding the relative contributions of different types of demographic changes, in isolation or conjunction. That it is simple is precisely what makes it powerful and portable from one problem to the next. Likewise, understanding how race and sex groups differ in terms of these features of grandparenthood is an important step in understanding intergroup inequalities in family structure and function.

## Electronic supplementary material

ESM 1 (PDF 204 kb)

## Footnotes

1 We refer to *grandparenthood* as having biological grandchildren (the biological children of one’s biological children). Although a nonsubstantial population in the United States today will experience step-grandparenthood (Yahirun et al. 2018), we are not studying that here.

2 Grandchild mortality can affect the duration of grandparenthood but rarely does.

3 We distinguish *childlessness* (having no living children) from *nonfertility* (not birthing or siring any biological children conditional on experiencing the age categories at which humans typically do so). In this distinction, childlessness can be caused by mortality—such as when someone dies before reaching childbearing ages—or nonfertility.

4 Code to produce the data files we analyze is available online (Verdery 2017).

5 Figures 3 and 4 do not plot observed outliers.

6 Mean or median trends in Figs 1–4 and 5–8 are comparable, but the scales are different, and Figs. 1, 2, 3, and 4 show means or medians over 10-year births cohorts whereas Figs. 5, 6, 7, and 8 show five-year moving averages of means or medians.
