# Supplementary material for: A Cohort Perspective on the Demography of Grandparenthood: Past, Present, and Future Changes in Race and Sex Disparities in the United States
Source: Demography. 2019 Jul 3;56(4):1495–518. doi: 10.1007/s13524-019-00795-1 (PMC6667684; doi:10.1007/s13524-019-00795-1)
Supplement: Supplementary file 1 — (PDF 204 kb) [file 13524_2019_795_MOESM1_ESM.pdf]

Appendix
"A Cohort Perspective on the Demography of Grandparenthood: Past, Present, and Future Changes in Race and Sex Disparities in the United States

Part A: Microsimulation Model Data Sources and Methods

We parameterize the microsimulation model with several data sources that reflect historical, contemporary, or projected demographic rates. As described below, we translate these rates into individual probabilities of demographic events at the monthly time scale. Appendix Table A1 illustrates some of the sources that we use, including input parameters that capture macro changes in demographic phenomena over the focal time period for the analysis, which are calculated from empirical data or other estimates. To assess the model's general fidelity to demographic history, this table also includes calculations of those same statistics from the resultant simulation data. Comparing the input data to the output data demonstrates that the model successfully replicates key features of demographic history in the United States, especially for more recent periods. Note that because the model begins in 1880 with an initially unmarried population, the output parameters for that year are highly biased as nearly all age eligible agents in that year attempt to marry; this distortion works itself out rapidly and is unlikely to bias our long run estimates.

The simulation begins in 1880 with 50,000 white agents and 50,000 black agents. Within race groups, the age and sex structure is proportional to the race-specific age and sex structure in the 1880 census because we drew each agent's attributes at random without replacement from that source (Ruggles et al. 2015). We focus on 50,000 agents in each race group because 50,000 is similar to what has been done in prior work (e.g., 40,000 people in Murphy 2011) and simultaneously large enough to offer statistically precise interpretations and small enough to maintain computational feasibility. Each agent then lives and dies according to monthly

probabilities of demographic events; along the way, agents might reproduces (creating other agents) or marry, divorce, or remarry with monthly probabilities also defining each of these events. We set probabilities of different demographic events according to different risk factors, most notably including year, age, sex, and race for all events but also, for some but not all events, including marital status and parity.

We define time-varying monthly probabilities of mortality by calculating race-age-sex-specific mortality probabilities. To do this, we rely on life-expectancy data, as shown in Table A1, which is one of the few available sources of information on changes in macro mortality conditions specified by race and sex over long periods. We use race-specific historical life expectancy data for the years 1880 to 1949 (Haines 2008), but unfortunately these data do not disaggregate by sex. To obtain sex-specific life expectancy estimates, we assume that the non-disaggregated statistics are midpoints and distribute male and female life expectancies around them such that women have higher life expectancy than men. Based on race-specific gaps in female compared to male life expectancy documented in available data from a historical but more recent period in the 1950s (NCHS 2016), we assume that white women had life expectancies one year higher than the non-disaggregated historical estimate and two years higher than white men, while black women had life expectancies half a year higher than the non-disaggregated historical estimate and one year higher than black men. Between 1950 and 2014, life expectancy estimates are disaggregated by race and sex, so we use those values (NCHS 2016). For estimating projected values, we rely on recent national projections from the United States Census Bureau (Colby and Ortman 2014); these are likewise disaggregated by race and sex. Across time periods, we linearly interpolate estimates for any missing years. To obtain age specific rates within race and sex groups, we use the updated Coale-Demeny Model West Life

Tables (U.N. Population Division 2015), which provide age specific mortality rates indexed by life expectancy levels. The SocSim microsimulation program translates age specific rates to monthly probabilities ("Demography/CEDA/PopCenter Microsimulation with SOCSIM" n.d.; Hammel et al. 1990)

To obtain monthly-age specific probabilities of fertility for women in each race group, we use a series of procedures that enable us to specify different probabilities for women of different parities and marital statuses. We start with data on the total fertility rate, which gives a general, time-varying indicator of the broader fertility conditions and which is available for long time periods disaggregated by race, as shown in Table A1 (sources: Haines 2008; Martin et al. 2015; NCHS 1999; U.S. Census Bureau 2012), with linear interpolations between years for missing cases. In the simulation, women older than 14 and younger than 51 can bear children. We construct different rates for women of three different parities – no prior births, one prior birth, and two or more prior births – and three different marital statuses – never married, currently married, and widowed/divorced. In each of the nine cross-classified parity and marital status categories, separately for women of each race, we define age-specific fertility probabilities. We do this by first fitting an ordinary least squares regression model to a standardized age-by-parity fertility data series for the United States drawn from the Human Fertility Collection (MPIDR & VID 2016). The original data decompose the total fertility rate into what levels are owed to women of different ages and parities; we standardize these into proportionate contributions. The coefficients from this model estimate the effects of age by parity by total fertility rate, and the regression fit the data well, predicted values from it close match the observed data, and adding additional control variables does not sufficiently increase model fit. We use the coefficients from this model to translate the time-varying total fertility rates from each race group into age by

parity rates; we added the minimum value to the very small proportion of cases where the series had a negative minimum predicted value. In the initial years of the simulation (1880-1900), we reaggregate the disaggregated age by parity specific rates into age-specific rates and use only the age-specific rates to make the model more computationally tractable; these procedures do not bias our long run estimates. In subsequent years, we use age by parity by marital status rates dynamically adjusted for population exposure, which we obtain by dividing the age by parity rates into the three marital status categories. We made this division to be consistent with the historical time series of the proportion of births for each race that occur to single, widowed, and married mothers as available in historical U.S. Census microdata (Ruggles et al. 2015); for projected values, we assume the continuance of recent trajectories. In total, a consequence of these procedures is that we assume that age by parity rates do not vary by race or marital status, but we believe that the advantage of being able to model increases in childlessness and marital as well as non-marital childbearing for women of different parities outweighs the limitations of this assumption.

For each birth, the simulation records who the child's mother and father are. When non-married mothers have a child, the simulation model selects a father from the unmarried population of men over the age of 15 in line and treats them what SocSim users refer to as cohabitating partners (Mason 2016). The simulation assigns each newborn child's sex at random in line with an input sex ratio at birth parameter. We obtain this parameter using empirical race-specific sex ratios at birth or estimates: from 1970-2002 we draw on direct estimates (Mathews and Hamilton 2005); from 1880-1970 we use race-specific averages in those data in the years 1970-1979, and from 2003-2060 we use race-specific averages in those data in the years 1993-2002.

Agents in the simulation have the opportunity to marry according to age- and sex-specific marriage rates. We assume that men have zero chance of marriage before age 16 and women have zero chance before age 15. We create time-varying, age-, sex-, and race-specific marriage rates from historical and projected estimates of two parameters: the singulate mean age at marriage and the of 45 year olds who ever married. We use different values for men and women and for blacks and whites; in the period 1880-2010 we use historical data for available years (Elliott et al. 2012) and linearly interpolate between years; for projected years we assume continuance of recent trends. To obtain age gradients in these rates, we first assume that about half of population members marry before the mean age at marriage (we calibrated exact proportions by race and sex to approximate observed trends). From those levels to age 45, we estimate race-sex-specific probabilities of marriage to fit the proportion of the group in question that is married by 45. From age 45 on, we set marriage rates for all race and sex groups to levels where those who are unmarried have probabilities of 0.005 of seeking a partner each month; someone who lived from age 45 to age 100 would have a 30% chance of marrying in this duration. We model remarriage by assuming that those who are divorced and widowed remarry at rates that differ by race and sex but are constant over time. Regardless of age or period, we use monthly remarriage probabilities that produce percentages of survivors remarried within 20 years that we base on historical estimates (Aughinbaugh et al. 2013; Livingston 2014) and our own model calibrations. Specifically, we use the following 20 year remarriage probabilities for survivors of the following race and sex groups: white men at 73% remarry over a survived 20 year interval, White women at 65%, Black men at 60%, and Black women at 45%. Finally, we allow agents to divorce according to divorce probabilities that we derive by combining multiple decrement life tables that provide anniversary schedules of divorce (Cohen 2016) and historical

estimates of crude divorce rates from 1880 to the 2010s (NCHS 1973, 2015) or our own crude future projections made by assuming that the United States crude divorce rate continues its recent trajectory of decline from the 2014 crude divorce rate (3.2 per thousand), but does so more slowly than it has of late such that it settles at 2.5 per thousand in 2060. We then linearly interpolate between the observed 2014 rates and the assumed 2060 rates. We adjust the anniversary schedule of divorce for each race and sex group by the historical and projected trends in crude divorce rates to obtain time-varying, anniversary, sex, and race specific divorce rates.

Part B: Comparison of Inputs and Outputs from Demographic Microsimulation Model

Table A1 presents the comparison of inputs and outputs from the demographic microsimulation model. Data are presented every twenty years from 1880 to 2040. The indicators shown are the total fertility rate, life expectancy for women and men, and the singulate mean age at marriage for women and men. We present input and output data separately for whites and blacks. There is a high degree of similarity across the input data to the simulations and the output data. The one exception is the mean age at marriage for men and women in the early cohorts of the simulation because the simulation begins in 1880 with a population of unmarried people, but by 1900, the data points line up very well.

Part C: Comparison of Simulation Data and Population Survey Data

Tables C1-C3 present comparisons between simulation estimates and population surveys. First, Table C1 presents the percentage with biological children. Note that among a set of surveys which are almost all designed to be nationally representative of the population across

similar time frames, there is substantial variability in the percentage of adults reporting biological children. First, we show that among the simulation sample in 2010, 80.8% of whites and 82% of blacks ages 50 and above had children. The other columns present comparable estimates from published papers using the Health and Retirement Study (Margolis & Verdery 2017) and the Panel Study of Income Dynamics (Daw, Verdery & Margolis, 2016), and the authors' own calculations using the General Social Survey (2010-2014), National Survey of Families and Households (1992-94), and International Social Survey Programme (2001). The simulation estimates are similar to the published estimates for similar age groups and periods. Table C2 presents comparison data on summary indicators of grandarenthood by race. By examining the data from the simulation and those estimated by the authors from the National Survey of Families and Households (1992-94), we can see that the percentage of adults with grandchildren and the median number of grandchildren among those with any, are similar. The median number of grandchildren among those with any are right on target. The percentage with grandchildren estimated from the NSFH show that whites are more likely to be grandparents than blacks, which shows a similar pattern in the simulated data. Last, Table C3 compares the percentage of older adults with grandchildren by age group and sex from the simulation and published estimates from Margolis and Wright (2017). This comparison shows men are less likely to be grandparents than women in all race and age groups across both data sources. In addition, as age increases, the percentage of adults with grandchildren similarly increases.

Appendix References

Aughinbaugh, A., Robles, O., & Sun, H. (2013). Marriage and divorce: patterns by gender, race, and educational attainment: *Monthly Labor Review* U.S. Bureau of Labor Statistics. http://www.bls.gov/opub/mlr/2013/article/marriage-and-divorce-patterns-by-gender-race-and educational-attainment.htm. Accessed 21 November 2016

Cohen, P. N. (2016). Multiple-decrement life table estimates of divorce rates. https://osf.io/zber3/. Accessed 21 November 2016

Colby, S. L., & Ortman, J. M. (2014). Projections of the size and composition of the US population: 2014 to 2060. US Census Bureau, 9.

Daw, J., Verdery, A. M., & Margolis, R. (2016). Kin count (s): Educational and racial differences in extended kinship in the United States. *Population and Development Review*, *42*(3), 491-517.

Elliott, D. B., Krivickas, K., Brault, M. W., & Kreider, R. M. (2012). Historical marriage trends from 1890-2010: A focus on race differences. Paper presented at the 2012 annual meeting of the Population Association of America, San Francisco, CA. https://www.census.gov/hhes/socdemo/marriage/data/acs/ElliottetalPAA2012paper.pdf. Accessed 11 November 2016

Hammel EA,Mason C,Wachter KW(1990) SOCSIM II, A SociodemographicMicrosimulation Program, Rev. 1.0, Operating Manual (Institute of International Studies, University of California, Berkeley).

Haines, M. (2008, March 19). Fertility and Mortality in the United States. In EH.Net Encyclopedia. Edited by Robert Whaples. http://eh.net/encyclopedia/fertility-and-mortality-in the-united-states/. Accessed 11 November 2016

Livingston, G. (2014). Chapter 2: The Demographics of Remarriage. Pew Research Center's Social & Demographic Trends Project. http://www.pewsocialtrends.org/2014/11/14/chapter-2-the-demographics-of-remarriage/.

Martin, J. A., Hamilton, B. E., Osterman, M. J. K., Curtin, S. C., & Matthews, T. J. (2015). Births: Final Data for 2013. (Hyattsville, MD., Vol. vol 64 no 1). National Center for Health Statistics. https://www.cdc.gov/nchs/data/nvsr/nvsr64/nvsr64_01.pdf [Accessed 2016-November-20].

Margolis, R., & Wright, L. (2016). Older adults with three generations of kin: Prevalence, correlates, and transfers. *Journals of Gerontology Series B: Psychological Sciences and Social Sciences*, *72*(6), 1067-1072.

Margolis, R., & Verdery, A. M. (2017). Older adults without close kin in the United States. *The Journals of Gerontology: Series B*, *72*(4), 688-693.

Mason C (2016) Socsim Oversimplified. Available at: http://lab.demog.berkeley.edu/socsim/CurrentDocs/socsimOversimplified.pdf [Accessed April 15, 2016].

Mathews TJ, Hamilton BE (2005) Trend Analysis of the Sex Ratio at Birth in the United States. Available at: http://www.cdc.gov/nchs/data/nvsr/nvsr53/nvsr53_20.pdf [Accessed November 11, 2016].

Max Planck Institute for Demographic Research (Germany) and Vienna Institute of Demography (Austria). Human Fertility Database. Available at www.humanfertility.org (data downloaded on 2016).

Murphy M (2011) Long-Term Effects of the Demographic Transition on Family and Kinship Networks in Britain. Population and Development Review 37:55–80.

NCHS. (1973). 100 years of marriage and divorce statistics, United States, 1867-1967. https://www.cdc.gov/nchs/data/series/sr_21/sr21_024.pdf. Accessed 11 November 2016

NCHS. (1999). Table 1-7. Total Fertility Rates and Birth Rates, by Age of Mother and Race: United States, 1940-99 - t991x07.pdf.

NCHS. (2015). NVSS - National Marriage and Divorce Rate Trends. http://www.cdc.gov/nchs/nvss/marriage_divorce_tables.htm. Accessed 21 November 2016

NCHS. (2016). Health, United States, 2015: with special feature on racial and ethnic health disparities. Hyattsville, MD: National Center for Health Statistics.

Ruggles, S., Genadek, K., Goeken, R., Grover, J., & Sobek, M. (2015). Integrated Public Use Microdata Series: Version 6.0 [Machine Readable Database]. Minneapolis: University of Minnesota.

Socsim (2016) Available at lab.demog.berkeley.edu/socsim/. Accessed April 15, 2016.

University of California, Berkeley (USA), and Max Planck Institute for Demographic Research (Germany). Human Mortality Database. Available at www.mortality.org or www.humanmortality.de (data downloaded 2017]).

U.N. Population Division. (2015). Model Life Tables for e0=20 to 100 by 1 year increment for ages up to 130 - the complete life tables. In World Population Prospects, The 2015 Revision.

U.S. Census Bureau. (2012). 2012 National Population Projections. https://www.census.gov/population/projections/data/national/2012.html. Accessed 17 November 2016

Table B1. Inputs and Outputs from demographic microsimulation model.

Year	Input into simulation model					Calculated from simulation model				
	TFR	e0, women	e0, men	SMAM, women	SMAM, men	TFR	e0, women	e0, men	SMAM, women	SMAM, men
	**Whites**									
1880	4.2	41.5	39.5	24	27	4.3	39.6	38.3	29	32
1900	3.6	52.8	50.8	24	26	3.7	50.6	49.3	24	26
1920	3.2	58.4	56.4	23	25	3.4	57.7	57.5	23	25
1940	2.2	65.9	63.9	23	25	2.3	67.7	61.6	23	25
1960	3.5	74.1	67.4	21	24	3.7	73.3	65.1	22	25
1980	1.8	78.1	70.7	23	25	1.9	77.0	70.2	23	25
2000	2.1	79.9	74.7	25	26	2.3	77.1	73.5	26	26
2020	1.8	82.9	78.0	27	29	2.0	82.7	76.7	26	29
2040	1.8	85.2	81.2	29	31	2.0	83.9	79.7	28	30
	**Blacks**									
1880	7.3	34.9	33.9	22	25	6.9	34.2	34.0	27	30
1900	5.6	42.3	41.3	22	25	5.6	43.5	41.4	22	25
1920	3.6	47.5	46.5	21	24	3.7	47.7	46.5	22	25
1940	2.9	54.4	53.4	22	24	3.0	54.0	53.2	22	25
1960	4.5	66.3	61.1	22	24	4.6	66.0	59.6	23	25
1980	2.2	72.5	63.8	25	26	2.2	72.3	64.1	23	25
2000	2.1	75.1	68.2	28	27	2.2	74.1	67.9	28	28
2020	2.0	79.8	74.0	31	32	2.1	78.0	72.9	30	32
2040	2.0	82.8	78.0	33	34	2.0	82.0	77.6	31	33

Table C1. Comparison of Simulation Estimates to Population Surveys: Percent with biological children

	Simulation (2010) Ages 50+ NH Whites	Simulation (2010) Ages 50+ NH Blacks	HRS (1998-2010) Ages 55+ All races	GSS (2010-14) Ages 50+ All races	NSFH (1992-94) Ages 50+ All races	ISSP (2001) Ages 50+ All races	PSID (2011) Ages 55+ NH Whites	PSID (2011) Ages 55+ NH Blacks
Percent with biological children	80.8	82.0	91.5	86.2	90.9	83.0	82.0	74.0

Notes: Health and Retirement Study (HRS) estimates come from Margolis and Verdery (2017) in *The Journals of Gerontology: Series B*. General Social Survey (GSS) estimates for biological children are based on those who never had children. National Survey of Families and Households is from wave 2 (NSFH). International Social Survey Programme (ISSP) data includes any siblings over 18 years old. Panel Study of Income Dynamics (PSID) estimates for NH Whites and Blacks are from Daw, Verdery & Margolis (2016) in *Population and Development Review*.

Table C2. Comparison of Simulation Estimates to Population Surveys: Summary Indicators of Grandchildren by Race

	Simulation (1990) Ages 50+ NH Whites	Simulation (1990) Ages 50+ NH Blacks	NSFH (1992-94) Ages 65+ Native-born NH Whites	NSFH (1992-94) Ages 65+ Native-born NH Blacks
Percent with grandchildren	74.9%	72.9%	79.7%	68.2%
Median number of grandchildren among those with any	5	6	5	6

Note: NSFH 1992-94 Wave 2, authors' own weighted calculations.

Table C3. Comparison of Simulation Estimates to Population Surveys: Percent with Grandchildren by Age and Sex

	Simulation (2010) NH White Women	Simulation (2010) NH White Men	Simulation (2010) NH Black Women	Simulation (2010) NH Black Men	HRS (1998-2010) All Races Women	HRS (1998-2010) All Races Men
Percent with grandchildren						
Age 60-69	76.6%	69.8%	80.0%	71.0%	85.7%	70.3%
Age 70-79	89.2%	83.0%	82.8%	80.7%	91.7%	86.5%

Note: Estimates from the Health and Retirement Study are from Margolis and Wright 2017 in *The Journals of Gerontology: Series B*.
